# Risk Factors for Typhoid Fever: Systematic Review

**DOI:** 10.2196/67544

**Published:** 2025-08-28

**Authors:** Portia Boakye Okyere, Sampson Twumasi-Ankrah, Sam Newton, Samuel Nkansah Darko, Michael Owusu Ansah, Eric Darko, Francis Agyapong, Hyon Jin Jeon, Yaw Adu-Sarkodie, Florian Marks, Ellis Owusu-Dabo

**Affiliations:** 1 School of Public Health College of Health Kwame Nkrumah University of Science and Technology Kumasi Ghana; 2 Department of Statistics and Actuarial Science College of Science Kwame Nkrumah University of Science and Technology Kumasi Ghana; 3 Department of Molecular Medicine College of Health Kwame Nkrumah University of Science and Technology Kumasi Ghana; 4 Community Health College of Health Kwame Nkrumah University of Science and Technology Kumasi Ghana; 5 Department of Clinical Microbiology College of Health Kwame Nkrumah University of Science and Technology Kumasi Ghana; 6 International Vaccine Institute Seoul, South Korea Republic of Korea; 7 Madagascar Institute for Vaccine Research (MIVR), University of Antananarivo Antananarivo Madagascar; 8 Department of Global Public Health, Karolinska Institutet Stockholm Sweden; 9 Heidelberg Institute of Global Health, University of Heidelberg Heidelberg Germany

**Keywords:** typhoid fever, risk factors, foodborne, waterborne, systematic review

## Abstract

**Background:**

Typhoid fever, a significant global health problem, demonstrates a multifaceted transmission pattern. Knowledge of the factors driving its transmission is critical for developing effective control strategies and optimizing resource allocation.

**Objective:**

This review aimed to comprehensively synthesize evidence on risk factors associated with typhoid fever transmission from 1928 to 2024.

**Methods:**

We searched PubMed, Scopus, Google Scholar, and Semantic Scholar databases using keywords related to risk, contributors, determinants, and causes of typhoid fever. We followed a registered protocol to support our search and triangulated the results.

**Results:**

Overall, we retrieved 1614 articles, of which 219 were reviewed. Of these, 109 addressed multiple, non–mutually exclusive typhoid fever risk factors. Unsurprisingly, of the total articles reviewed on risk factors, approximately 70.6% (77/109) originated from the Asian continent (51/109, 46.8%) and the African continent (26/109, 23.9%). Half of the articles (55/109, 50.5%) focused on risk factors related to demographic and socioeconomic transmission, while 44% (48/109) of the articles examined foodborne transmission. Additional risk factors included water, sanitation, and hygiene practices: waterborne transmissions (45/109, 41.3%) and sanitation and hygiene practices (34/109, 31.2%), travel-related risk (19/109, 17.4%), antimicrobial use (14/109, 12.8%), climate-related factors (15/109, 13.8%), environment-related factors (9/109, 8.3%), typhoid carriers (11/109, 10.1%), and host-related risk factors (6/109, 5.5%).

**Conclusions:**

This review identifies demographic and socioeconomic factors as key drivers of typhoid transmission, underscoring the need for targeted interventions. Strengthening street food regulation in urban Asia and investing in water infrastructure in rural Africa can significantly mitigate risk. Integrating water, sanitation, and hygiene interventions with typhoid vaccines can reduce immediate exposure while enhancing long-term immunity. Prioritizing these strategies in schools and high-risk communities is essential for sustainable typhoid control. Future research should focus on longitudinal studies to assess risk factor causality and vaccine impact, guiding more effective public health interventions.

## Introduction

### Background

Typhoid fever is a potentially fatal febrile systemic disease caused by *Salmonella enterica* serotype Typhi (*Salmonella* Typhi or *S* Typhi), a rod-shaped gram-negative bacterium belonging to the Enterobacteriaceae family. *S* Typhi exists exclusively in humans and causes illnesses (typhoid fever) that resemble many other febrile diseases [1]*.* In this study, the terms “typhoid fever” and “typhoid” are used interchangeably. A description of the infection was reviewed by Cunha [2], clearly separating it from other febrile illnesses and associating its clinical manifestation with significant pathological abnormalities in the spleen, mesenteric lymph nodes, and intestines. Nonetheless, the mainstay of diagnosis is a microbial culture, usually with blood or bone marrow samples. Although bone marrow culture is highly sensitive, it is both invasive and technically unfeasible in most settings. As a result, the disease is usually diagnosed with blood culture, despite its limited sensitivity of approximately 40% to 80%, partly due to antibiotic exposures before the patient visits the health facility [3].

The disease transmission is by the fecal-oral route and can take 2 main forms: direct transmission, where food and water in the immediate environment are contaminated through poor hygiene and sanitation practices, either by transient or chronic carriers; and indirect transmission, where the broader environment becomes contaminated when sewage pollutes water supplies, raw human feces or untreated sewage is used as fertilizer for crops, or piped water is inadequately treated [4].

Typhoid fever is reported to affect people of all ages, although children are more susceptible than other age groups [5]. Before 2000, the global burden of typhoid fever was estimated at 16 million illnesses and 600 thousand deaths annually [6]. In 2000, approximately 21.7 million illnesses and 216,000 deaths occurred [7]. By 2010, annual estimates indicated approximately 26.9 million cases and 200,000 fatalities [8]. However, a more recent estimate from 2017 to 2024 suggests a decline in the annual incidence of typhoid cases [9,10]. Despite this decrease, typhoid fever remains a significant public health concern, particularly in areas with limited access to clean water and sanitation. Typhoid fever can be prevented and controlled concurrently with vaccinations and advancements in food safety, water quality, hygiene, and sanitation [11]. Three main generations of typhoid vaccines are presently approved for use by the World Health Organization (WHO): typhoid conjugate vaccines (TCVs), live attenuated Ty21a, and the unconjugated Vi polysaccharide vaccines [4]. The WHO strongly recommends using TCVs for all ages due to their superior immunological properties, suitability for use in younger children, and predicted longer period of protection above 2 years, which was a major limitation for using the Vi polysaccharide. However, to inform the choice of vaccination in a country, evidence is needed on both the scope of the problem and the risk factors contributing to disease transmission [11]. Despite notable progress in typhoid control, the disease remains a significant cause of morbidity and mortality to which billions of people worldwide are continuously exposed, particularly in Asia and sub-Saharan Africa.

Typhoid fever susceptibility involves multiple factors, each contributing through distinct transmission pathways. In endemic countries, knowledge of typhoid fever risk factors is critical for developing effective control strategies and allocating resources. Several epidemiological and modeled studies [12-14] have explored location- and time-specific risk factors for typhoid fever. In addition, various review studies have been undertaken to comprehensively understand and address the risk of typhoid across different transmission routes. For instance, Lee et al [15] used geospatial modeling to develop a typhoid risk index based on factors such as water sources, toilet facilities, and population density, providing insight into the geographical distribution of typhoid risk in impoverished countries. Similarly, Kim et al [16] investigated the relationship between observed incidence rates and geospatial covariates, such as access to improved water and sanitation, as well as broader health and environmental conditions influencing the transmission of *S* Typhi. Furthermore, Brockett et al [17] systematically reviewed case-control studies to uncover associations between water, sanitation, and hygiene (WASH) practices, food exposures, and typhoid fever. Similarly, Mogasale et al [18] conducted a meta-analysis spanning 1990 to 2013 to estimate the risk of typhoid associated with inadequate access to safe water. In addition, Wang et al [19], in a systematic review and meta-analysis, described the patterns of salmonellosis outbreaks in China from 1970 to 2023. Other reviews have examined specific aspects of typhoid transmission. For instance, the study by Ma et al [20] reviewed human genetic variants affecting susceptibility to enteric fever infection, while Levantesi et al [21] assessed the contribution of natural freshwater and drinking water as routes of *Salmonella* contamination from 2000 to 2010.

### Objectives

While these studies provide valuable insights into different aspects of typhoid risk, a holistic synthesis of socioeconomic, environmental, and other factors remains lacking. Furthermore, previous reviews have often been limited in temporal scope or focused on specific transmission pathways. To address this gap, this study examined typhoid risk factors across a broader time frame (1928-2024) and incorporated a multidimensional perspective on transmission dynamics. By systematically integrating evidence from diverse sources, we aimed to provide a more comprehensive understanding of typhoid fever risk factors, which can inform targeted interventions to reduce typhoid incidence worldwide.

## Methods

### Search Strategy

We searched PubMed, Scopus, Google Scholar, and Semantic Scholar databases for articles published on risk factors for typhoid fever. The search was conducted in June 2023, and titles and abstracts from databases were downloaded and saved. Each database was searched using the following terms and keywords: risk factors, factors, contributors, determinants, causes, predictors, susceptibility factors, factors of exposures, predisposing factor, typhoid fever, typhoid, *Salmonella* typhoid, *Salmonella* Typhi, *S* Typhi, typhoid disease, typhoidal salmonellosis, and typhoidal *Salmonella*, and searches excluded terms related to perforation, complication, virulence, severity, and nontyphoidal infections. We placed no restrictions on the publication year, but the language was restricted to English. We followed a protocol adapted from the PRISMA (Preferred Reporting Items for Systematic Reviews and Meta-Analyses) guidelines, which was registered with the Open Science Framework in January 2024 [22] to structure our search. See Multimedia Appendix 1 for the PRISMA checklist. This study used published articles, and as such, permission from the institutional review board was not required.

### Study Screening and Selection Criteria

We screened and selected studies on typhoid fever risk factors based on the inclusion and exclusion criteria summarized in Textbox 1.

**Inclusion and exclusion criteria for the desk review search strategy on typhoid fever risk factors.**

**Inclusion criteria**

- Studies with confirmed S Typhi infections or outbreaks identified through the following: culture of bodily fluids or stool; polymerase chain reaction; Widal or other serological methods
- Epidemiological or modeled studies of any design

**Exclusion criteria**

- Studies on severe infections (complications and mortality)
- Studies that classified typhoid fever solely based on clinical indicators (ie, signs and symptoms) or with unclear diagnostic methods
- Studies involving nonhuman participants (animals, water, and farm produce)
- Articles whose full text was not available in English or those inaccessible

Further details on inclusion and exclusion criteria can be found in the review protocol [22].

The titles and abstracts retrieved from each database were imported into EndNote X8.2 (build 13302), merged into a single reference list, and duplicates were eliminated. The deduplicated list was then uploaded to a web-based systematic review tool, Rayyan (Qatar Computing Research Institute) [23] for title and abstract screening. All included citations were exported into Microsoft Excel (version 16.16.27) for full-text retrieval and screening. Each subsequent process, including title and abstract review, full-text review, and data extraction, was performed using predefined screening guidelines outlined in the screener instruction section of the review protocol [22] to ensure consistency. One author (PBO) performed the initial screening with supervision from coauthors (EO-D, SN, and ST-A). Discrepancies in the study selection were resolved through discussion among authors (PBO, EO-D, SN, and ST-A), with unresolved cases adjudicated by EO-D. Additional relevant articles were identified through other sources (expert input) and included after being assessed using the same eligibility criteria. Data were then extracted into Microsoft Excel and a shared Google Sheets spreadsheet (Google LLC; Multimedia Appendix 2). All authors reviewed the final dataset for completeness and accuracy.

### Data Extraction

Electronic searches were performed using the internet to locate all eligible articles, and all relevant data relating to the research question were manually extracted into Microsoft Excel after reading the full text. The extracted data included specific risk factors for typhoid fever in all eligible articles. In addition, data on the route of transmission, sources of infection, year of publication, data collection period, town or district, country and continent of the study, study setting (outbreak or endemic), diagnostics method, study type, number of *S* Typhi cases, total participants enrolled, ages of participants, study design, and citations were extracted (Multimedia Appendix 2). We grouped the ages of participants into 3 categories based on inclusion age and age ranges: “children” were ≤15 years, “adults” were >15 years, and “mixed ages” were both children and adult participants. Information on typhoid fever susceptibility was grouped according to their transmission routes: waterborne and foodborne transmissions, host risk factors, vaccination, travel-related risk, health education, occupational risk, population growth and overcrowding, sanitation and sewage systems, climate and meteorological factors, antimicrobial resistance factors were extracted.

## Results

### Overview

**Figure 1:**
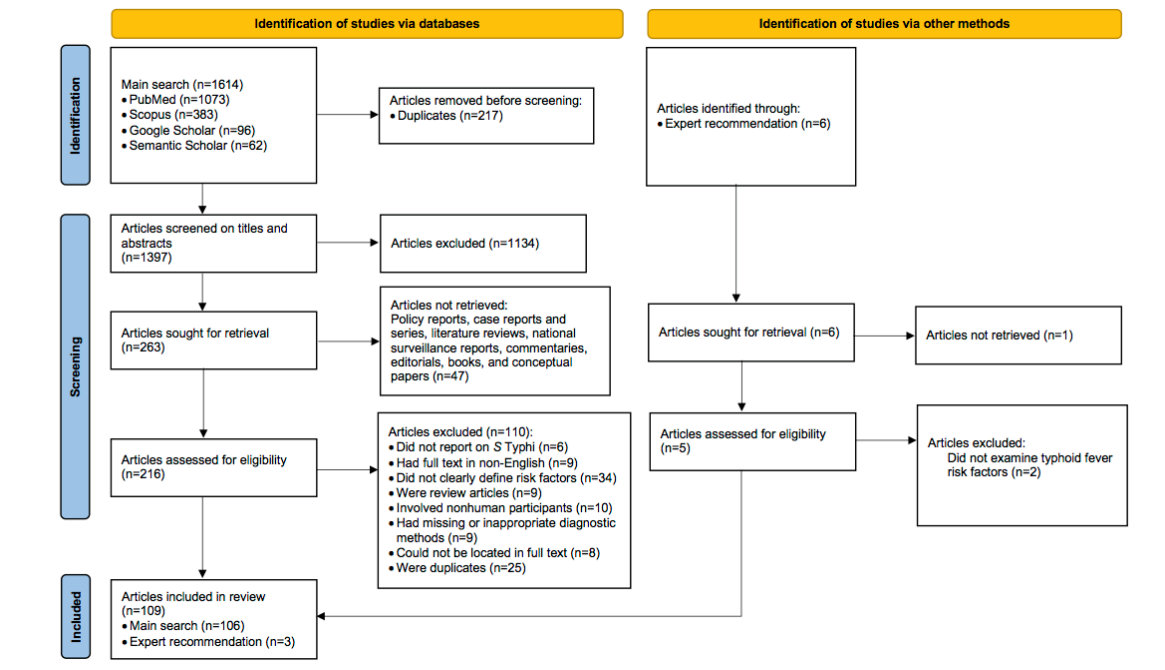
PRISMA (Preferred Reporting Items for Systematic Reviews and Meta-Analyses) flow diagram of search strategies and article selection of risk factors for typhoid fever (2024).

Our search strategy initially identified 1614 articles published between 1928 and 2023. After removing 217 duplicates, 1397 titles and abstracts remained for screening. Of these, 1181 were excluded, with the majority (687 articles) not examining typhoid fever risk factors. After a full-text review of 219 articles from the main databases (216 articles) and expert recommendations (3 articles). Other reasons for exclusion included 25 duplicates of the same study published by different authors in different journals; 10 studies involving nonhuman participants such as farm-produced and water samples; 17 articles with non-English full texts or unavailable full texts; 6 articles not specifically related to *S* Typhi; and 9 articles with missing or inappropriate diagnosis based on recall typhoid fever episodes, unclear diagnosis, and clinical indicators (signs and symptoms). Furthermore, to avoid content duplication, 9 review articles were excluded, as shown in Figure 1. Finally, 109 published articles were included in this study [12-14,24-128] (Multimedia Appendix 2).

### Study Characteristics

Among the 109 eligible articles, data were extracted from publications spanning 1972 to 2024 (Multimedia Appendix 2), covering 6 continents: 24% (26/109) from Africa, 46.8% (51/109) from Asia, 0.9% (1/109) from South America, 8.3% (9/109) from North America, 10.1% (11/109) from Europe, and 6.4% (7/109) from Australia. Furthermore, 2.8% (3/109) of the articles collected data from mixed continents, while one article (1/109, 0.9%) relied on the GeoSentinel Surveillance Network database, lacking specific location details (Table S1 in Multimedia Appendix 3). Regarding transmission routes, multiple non–mutually exclusive typhoid risk factors were identified across the eligible articles (Table 1) for both common and specific risk factors. Waterborne transmission was reported in 41.3% (45/109) of the articles, while 44% (48/109) of the articles focused on foodborne transmission. Demographic and socioeconomic factors were identified in more than 50.5% (55/109) of the studies, with hygiene and sanitation discussed in 31.2% (34/109) of the studies. Additional risk factors included travel-related exposure (19/109, 17.4%), climatic influences (15/109, 13.8%), and antimicrobial use (14/109, 12.8%; Table S2 in Multimedia Appendix 3). Across 103 papers, 253,951 typhoid fever cases were reported, with a median (IQR) of 110 (51-283). The diagnosis was confirmed predominantly through culture in 84.4% (92/109) of the articles, with approximately 9% (10/109) of articles using Widal and other serological tests and 6.4% (7/109) of the articles using polymerase chain reaction and other sequencing techniques. Age distribution data were available in 103 articles. Most of the articles (83/103, 80.6%) included participants of mixed ages, while approximately (14/103, 13.6%) of the articles focused exclusively on those aged ≤15 years and 5.8% (6/103) of the articles focused on those aged >15 years (Table S1 in Multimedia Appendix 3).

**Table 1:** Common and specific risk factors for typhoid fever.

Common risk factors		Specific risk factors
**Waterborne transmission**		
	Water sources	Household sources of drinking water [24,58], having multiple drinking water sources [68], using water from other sources than the municipal water networks for bathing, brushing teeth or drinking [112], primary water sources with unpleasant smell [37], obtaining water from an outside tap [46], unsafe water source [53,115], obtaining water from municipal pipe for drinking [107], obtaining water from a river or stream [12,115]
	Water supply	Water supplied by an outdated gravity-fed network [53], intermittent water availability [12], inadequate safe water supply [14], defective water systems [113], household water supply from public wells and boreholes or merchants [98], water from a community [34], water from government overhead tanks [43]
	Contaminated or unsafe water	Drinking unsafe or contaminated water [28,33,63,79,95], using substandard water [83], *Escherichia coli* in stored drinking water [79], water sold in small plastic bags [80], use of ice cubes from a street vendor [29,123]
	Untreated water	Drinking water from a well [96], untreated household drinking water [37,46-48,70,120], use of untreated public water after rains [32], drinking water from untreated open sources [12,27,89], drinking water at the work site [34], use of bore water [107], accidental ingestion of contaminated river water during swimming or bathing [28], cooking and cleaning with river water or an open dug well [68]
	Water storage	Storing water in plastic containers without a lid [98], not storing water for drinking in a narrow-mouthed container [38], not using tipped containers to draw water [38], water sold in small plastic bags [80]
**Foodborne transmission**		
	Street stall or restaurant food	Eating out from commercial food stalls, restaurants, or mobile food vendors in the street or outside home [26,34,45,91,101,122], eating outdoors at least once a week [29,30,117], eating food from a roadside cabin during the summer [34], mothers eating food from street vendors [70], eating cold beverages outside home [45], not dining at a tea-house [47], consuming French fries with sauce and poppadum from street vendors [107], eating commercially available foods or drinks [120], eating food at community market [97]
	Poor food hygiene	Eating unwashed farm produce [12], unwashed guavas [38], not washing vegetables before eating [47]
	Food handlers	Consuming food items from vendors [108,122], male food handlers [40], consuming food with the mother’s or caregiver’s assistance [30], drinking orange juice with hand contact [50], poor or unhygienic food handling practices or procedures [14,100], eating food prepared at home [97]
	Salads or other contaminated food	Eating contaminated foods [63], including cucumber salad [94], potato salad [114], lettuce salad [31], and raw salads (onion, cucumber, and tomato) [103]
	Uncooked or raw food	Eating raw traditional foods such as cig kofte [31], raw onions and cabbage [38], raw milk and meat [58], uncooked shellfish [118], papaya [37], and unwashed guava [38]
	Frozen food	Eating ice cream [30,34,49], ice cubes in beverages [29], frozen tropical fruit (mamey) shakes [84], and fresh ice cream during the hot season [13]
	Milk products	Eating butter and yogurt [38], fresh yogurt made from cow and sheep in the summer [13], and homemade cheese [13]
	Local or traditional food	consuming locally made beverages [80], eating locally prepared popsicles [103], and consuming locally prepared flavored beverages [103]
	Other	Eating food brought by relatives from endemic areas [91], sharing food from the same plate [123], and sharing food plates [53]
**Demographic, socioeconomic, hygiene, and sanitation factors**		
	Demographic background	Age [25,58,87,126], older age [39,86], younger age [33,42,59,82,108,109], sex of the individual [102,126], being female [108,121] or male [87,101], presence of preschool children in the household [103], young adult [121], demographic status [56], and low BMI [128]
	Socioeconomic status	Poor or low socioeconomic status [25,56,76,83,104], attending a gathering [96], per capita disposable income of all residents, and per capita gross domestic product [102], unemployment or part-time work [29,128], being part of a nuclear family [49], wealth index [90], being a student [47], and attending school or daycare [68]
	Education level	No or low educational level [27,48,120], educational level [58], years of schooling [90], students in conventional institutions of higher learning [102], and illiteracy rate [49,104,105]
	Occupational risk	Mishandling of *S* Typhi samples by clinical microbiology laboratory staff [72]; medical and laboratory personnel and sewage workers occupationally exposed to *Salmonella* bacteria [91]; household member growing crops [68]; farmers [87,128]; rearing chicken or goats [92]; job-related cause [63]
	Population growth and overcrowding	Rising or bigger household size [104,127], living in a crowded household [31], crowding poor living conditions [63], and increased population density [33,113]
	Housing system or condition	Poor housing conditions [79]
	Hygiene and behavioral factors	Scarcity of soap near a hand washing facility [127], nonuse of soap for handwashing [12,49,69,108], nonuse of medicated soap [127], nonavailability of soap to wash hands after toilet use [68,104], a habit of not washing hands before cooking or after defecating [53], infrequent hand washing after latrine use [12,97], poor hand washing practices [105], occasionally or never washing hand with water and soap [26,122], never or rarely washing hands before preparing or handling food, and eating or feeding [29,30,120]
	Water, sanitation, and hygiene (WASH) practices	Poor WASH practices [74], not living in a better WASH household [75,106]
	Sanitation and sewage systems	Use of pit latrine [27,92], open defecation [92], improper disposal of solid waste [27], burst sewer pipes at home [96], living in houses with open sewers [29], visible urine or feces [53], poor sanitary practice [14,63,95,98,113], having home latrines [38], no toilets in the residence [108,115], poor excreta disposal [79], having unimproved or malfunctioning sanitation infrastructure [12], unsterilized water from the hospital disposal and residential sewage used to irrigate vegetable farmlands [110], inadequate public sewerage system [90], and poor toilet drainage soil [79].
**Other**		
	Antimicrobial use	Frequent use of antimicrobials or history of antimicrobial use [34,47,55,62,63,76,81,101], chloramphenicol-resistant *S* Typhi strain [44,79], ceftriaxone-resistant *S* Typhi strain [119], multidrug resistant or extensively drug-resistant *S* Typhi strains [121], circulation of virulent *S* Typhi strain (H58-lineage) [60,103]
	Host risk factors	Polymorphism in intronic variable number tandem repeat of IL-4 [35], presence of serum anti–*Helicobacter pylori* immunoglobulin G antibodies [49,109], history of chronic underlying disease [69], HIV infections [71], and haplotype of tumor necrosis factor locus from single nucleotide polymorphisms [124]
	Typhoid carriers	Recent or close contact with a patient with confirmed or active typhoid fever [32,48,63,88,101,105], hospitalization of household member with febrile illness [68], history of typhoid fever infections [69], having typhoid carrier at home [65], recent typhoid fever case in the household [108], and having a housekeeper (a boy or girl) [120]
	Vaccination	No or lack of vaccination [63,75,106], vaccine hesitancy [120], vaccine ineffectiveness [94], and poor vaccination coverage [103]
	Health education	Lack of knowledge regarding typhoid fever contact [127] and poor awareness of typhoid fever disease [97]
	Travel-related risk	Longer duration of stay in the endemic area [39]; returning from or visiting endemic countries [76,86,116,126]; visiting friends and relatives in endemic areas [39,73]; travel destination [64]; travel outside the United States, Sweden, or United Kingdom (international travel) [41,59,93,129]; Asian travelers [52]; children visiting friends and relatives in endemic places (particularly South Asia) [61,99]; recent travel to endemic areas [85,125]; transient male workers [82]; living in a metropolitan area [86]; urbanization [90]; and number of foreign tourists received (tourism) [102]
	Environmental conditions or factors	Living in geographically lower elevation areas [42], neighbors to a typhoid fever case [54], potentially floodable areas [57], proximity to major rivers and creeks [57], housing (external condition) [79], a lack of agricultural land [115], hydrological catchment areas [119], residing closer to waterbodies, residing near typhoid study treatment centers [104], anthropogenic alteration of land cover and hydrology [78], and environmental factors [56]
	Climate or meteorological factors	Seasonal variation or fluctuations [25,82,102,110], high temperatures during summer [103], rainfall [57,115], temperature and precipitation [111,115,126], high vapor pressure [115], rainy and harmattan seasons [36], extreme weather conditions [77], higher or hot temperatures [66,86], flooding [87], and wind speed [90]

## Discussion

### Principal Findings

This study synthesizes typhoid fever risk factors from 1928 to 2024; however, the included studies span from 1972 to 2024, as few earlier publications met the inclusion criteria. We have identified demographic and socioeconomic factors as the predominant pathways for typhoid fever transmission, with additional contributions from waterborne and foodborne routes, hygiene and sanitation, travel-related exposures, antimicrobial use, and typhoid carriers. These findings highlight the multifaceted nature of typhoid fever transmission and underscore the importance of targeted interventions. This evidence is crucial for clinicians, public health experts, and policy makers in designing effective control strategies and optimizing resource allocation, particularly in endemic regions.

Approximately 70.6% (77/109) of the reviewed articles collected data from Asia and Africa, where most developing countries are located. This aligns with the global burden of typhoid fever estimation, which shows that Asian and African countries bear the greatest burden [130]. The low number of articles in Europe, North America, and Australia can be attributed to the introduction of control programs such as the treatment of municipal water, pasteurization of dairy products, and strict food safety regulations [1,131]. Similarly, the lack of studies from South America can be attributed to the decline in typhoid burden, given the economic transition with improved water and sanitation in the area [1]. Despite the lower disease burden in these underrepresented regions (Europe, North and South America, and Australia), the risk factors identified in this study, such as socioeconomic, foodborne and waterborne transmission, and antimicrobial use, remain relevant to these regions. Emerging threats, including climate change, urbanization, and increased global travel, may contribute to the reemergence of typhoid in areas where it was previously controlled. In addition, the rise of antibiotic-resistant strains presents an ongoing challenge, underscoring the need for public health preparedness in these regions.

### Waterborne Transmission

Water sources greatly impact the spread of typhoid fever, with protected wells and piped water classified as safe, while rivers, streams, and other unprotected sources are deemed unsafe [132]. This study identified several specific risk factors associated with typhoid fever and water sources, including having multiple drinking water sources [68], use of nonmunicipal water sources for various purposes [29,68,107], and main water sources with a foul smell [37]. Households with access to multiple water sources may be at increased risk of typhoid fever, particularly when they face challenges with potable water access such as limited supply hours, high tariffs, low-pressure, and long distances to collection points. These challenges often impede their ability to meet daily needs. Consequently, some households, particularly those located close to open dug wells, rivers, or streams, may use these alternative sources for bathing, cooking, or even drinking [115]. Although such sources offer convenience, they often lack chlorination and may be contaminated with fecal matter, thereby posing a risk for typhoid fever transmission.

A water supply system defect can facilitate the transmission of typhoid fever. Our study discovered a typhoid fever outbreak associated with a gravity-fed network [53], indicating a probable spread through an outdated mains system. This was attributed to low water pressure, insufficient chlorination, and fecal infiltration [12].

Furthermore, we uncovered an intermittent piped water supply as a risk factor for typhoid transmission. Intermittent piped water supply is a common challenge in many developing countries, potentially compromising water supply quality by allowing contaminants in nonpressurized pipes and creating negative pressure conditions that enable pathogens such as *S* Typhi to infiltrate the system [12,133]. Such interruptions often compel households to rely on water storage, which may introduce additional contamination risks, and to seek alternative sources that may not be microbiologically safe [12,29]. Sources such as government overhead tanks [43], community or public taps, and protected wells [34,115], which are often considered safe, can become contaminated due to environmental exposures, including industrial activities, sewage discharges, agricultural runoff, and animal waste [28,80]. Under these conditions, the presence of *Escherichia coli* (*E coli*) in drinking water is commonly regarded as an indicator of fecal contamination and, by extension, a potential risk factor for typhoid fever due to the possible presence of enteric pathogens [79]. However, the relationship between *E coli* and typhoid fever risk remains inconclusive. For instance, Karkey et al [134] observed a link between high *E coli* concentrations and the presence of *S* Typhi nucleic acids in drinking water, suggesting that *E coli* contamination may serve as a proxy for typhoid transmission risk. In contrast, Luby et al [34] found no significant difference in the levels of *E coli* in water samples between households with typhoid cases and control households, challenging the consistency of this connection. These discrepancies may stem from environmental conditions, regional differences in water treatment practices, and microbial competition. For instance, in settings with inadequate chlorination, *E coli* presence may serve as a proxy for recent fecal contamination, thereby increasing typhoid risk. Conversely, in areas with intermittent water supply and biofilm formation within pipes, *S* Typhi may persist independently of *E coli*, complicating its use as a universal indicator.

In addition, water storage practices emerged as a risk factor for typhoid fever in our study. Evidence suggests that the microbiological quality of water often deteriorates after collection, particularly during storage and handling [98]. The risk of contamination is influenced by the type of vessel used for storing or drawing drinking water. Wide-mouthed containers, in particular, are associated with a higher risk of infection due to their larger open surface area, which increases exposure to environmental contaminants and the likelihood of fecal contamination, compared to narrow-tipped or covered containers [38,98].

Moreover, the use of untreated water, both inside and outside the home, poses significant risks for typhoid fever transmission [27,32,48,89]. Although municipal water systems may incorporate filtration and chlorination, cross-contamination with *S* Typhi through wastewater intrusion remains a concern [46]. In such contexts, household-level water disinfection may be necessary to enhance water quality [37]. However, some households opt out of water treatment due to the perceived reliability of their water sources [98]. While water from piped or otherwise protected sources may contain insufficient bacteria to cause typhoid fever, untreated water from unprotected sources may carry high *S* Typhi levels, sufficient to cause clinical disease [28,34,47]. All the aforementioned risk factors are listed in Textbox 1.

### Foodborne Transmission

Food serves as a highly efficient medium for the growth of *S* Typhi compared to water [34]. This study revealed specific risk factors indicating potential foodborne transmission of typhoid fever, with street stalls (restaurants) emerging as a major factor in this category. Street food stalls are typically small, with outdoor seating and without refrigerators or easy access to potable water or adequate facilities for washing food and utensils [29,34,45]. Therefore, persons who frequently consume food from such establishments are at increased risk of developing typhoid fever [29]. This increased risk may stem from using untreated or tap water stored or served in contaminated containers for food preparation and drinking purposes [45]. In addition, poor hygiene practices among street food vendors, including irregular handwashing [122], and the potential exposure to carriers of *S* Typhi further contribute to the risk of infection [135]. In addition, many of these food preparers and handlers in the street eateries lack adequate knowledge of safe food handling practices necessary to avert *S* Typhi infection transmission. Often, they operate without licenses or registration from food safety authorities; as a result, they are neither trained nor subject to regular inspections. Consequently, they may unknowingly share food and drinks using poorly cleaned cups and utensils among multiple clients [97,120]. Furthermore, this study discovered a relationship between the consumption of frozen foods, including ice cream [13,30,49], fruit shakes [84], and iced beverages [29], and typhoid fever. A potential source of contamination lies in the ice used by street vendors, who often purchase large blocks of ice produced from untreated water, typically intended for industrial use, such as fisheries, rather than for human consumption. Despite this, the ice is commonly served in drinks for customers. Notably, research has shown that *S* Typhi can survive in ice for extended periods, underscoring its potential role as a vehicle for transmission [29,45,122]. Moreover, iced drinks may be further contaminated by street vendors who are asymptomatic carriers of *S* Typhi during the distribution chain [29]. In contrast, dining at tea houses decreases the risk of typhoid fever, as customers are typically served boiled water and tea, practices that limit exposure to *S* Typhi through thermal inactivation of the pathogen [47]. In addition, consumption of uncooked or raw foods such as onions, milk, meat, shellfish, papaya, cabbage, and other traditional raw ingredients poses a considerable risk, as these items may harbor *S* Typhi if not properly handled or sourced [31,37,38,58,118]. For instance, failure to wash fruits and vegetables before consumption increases the risk of infection due to surface contamination [12,38,47]. Cross-contamination during meat handling is another concern; for example, using the same knives and cutting boards for both infected and uninfected meat in butcheries and restaurants. Similarly, if contaminated water is used to wash carcasses or clean food-contact surfaces, *S* Typhi may be introduced during processing [58]. Moreover, inadequate hygiene practices during milk processing may contribute to the contamination of dairy products, including butter, yogurt, and homemade cheese [13,38,58]. Although dairy animals do not harbor *S* Typhi [136], improperly handled dairy products can serve as effective growth media for the pathogen [58].

### Demographic and Socioeconomic Factors

This study identified mixed findings regarding sex-based susceptibility to typhoid fever. While a study suggests that men face greater exposure due to occupational differences, mobility patterns, dietary factors, or a lack of immunity [82], other studies indicate that women, influenced by their physiological status, hormonal imbalance, and gender-specific activities, are more susceptible [120,121]. Building on this, we propose the hypothesis that occupational exposure may explain the higher risk in men, whereas caregiving roles could contribute to increased susceptibility among women. Nonetheless, a study by Rasul et al [137] concludes that typhoid fever incidence is independent of gender, affecting men and women equally across all age groups. Age serves as a significant factor in typhoid transmission, with both young children and older adults identified as vulnerable groups [121,138]. Young children, characterized by their underdeveloped immune systems [33,42,87] and a limited understanding of disease transmission [58,87], face heightened risks of infection. While younger adults are predisposed to infections due to their adventurous lifestyle or unsanitary activities, such as eating junk food, and an increased number of social gatherings [25,42,58], older adults are more likely to be *S* Typhi resistant due to continual immune boosting [138]. Conversely, older adults may experience susceptibility due to a waning immune system or increased exposure to occupational and environmental risks. These exposures may include involvement in farming-related water contact activities [58,68], rearing chicken or goats [68], handling *S* Typhi specimens in clinical settings [72,91], and working in sewage management [91]. Although *S* Typhi is a human-adapted pathogen and not naturally harbored by animals, the association with poultry and livestock rearing may act as a confounder, reflecting underlying poor sanitary and hygienic conditions within the household rather than direct transmission from animals [92].

Our investigation further revealed that socioeconomic status correlates with an increased likelihood of *S* Typhi infection [96]. While studies suggest that typhoid fever is more common in low-income countries and is connected to poor public health and low socioeconomic indicators [25,83], one study [90] identified a protective effect of the wealth index. This suggests that residing in affluent districts within low- or middle-income countries may significantly mitigate the risk. This may be attributed to the per capita gross domestic product and individual disposable income within a community [102]. Wealthier households are more likely to afford preventive measures such as clean drinking water, improved sanitation, and timely medical consultations, thereby reducing the risk of infection. Conversely, individuals in lower socioeconomic brackets often face health care barriers, leading to underdiagnosis and delayed treatment. The protective effect of a higher wealth index underscores the need for targeted interventions. Public health strategies should prioritize health care accessibility and sanitation improvements in economically disadvantaged areas. Investments in water and sanitation infrastructure, subsidized vaccination programs, and awareness campaigns tailored to low-income communities could help reduce the disproportionate burden of typhoid fever. In addition, strengthening diagnostic capacity in resource-limited settings can improve case detection, ensure timely treatment, and curb typhoid transmission while enhancing health outcomes.

In the context of education, our research highlights the lack of certificate education as a significant risk factor influencing the perception of typhoid fever. Individuals who have never attended school tend to possess limited knowledge about the disease and its modes of transmission, highlighting a strong link between lower educational attainment and reduced awareness [102,120]. Previous studies [90,104,105] emphasize that formal education increases understanding, with uneducated individuals more likely to contract typhoid fever. Interestingly, being a student [47] or attending school (daycare) [68] may also pose risks, likely due to certain exposures in educational settings. While essential health knowledge, such as WHO-recommended practices of handwashing with soap, can be acquired outside formal education, a general lack of awareness significantly increases the risk of infection. Individuals who do not recognize the risks are less likely to take preventive actions [97,127]. Another significant factor for typhoid transmission, besides knowledge, is awareness of the presence of a patient with typhoid at home [120]. Individuals who are unsure of the presence of a patient with chronic or current typhoid at home are more likely to have typhoid or a recurrence than those with full awareness. This may be attributed to the continued shedding of *S* Typhi in the stool and urine of infected individuals, even after initial antimicrobial treatment. Up to 10% of patients may continue shedding the bacteria for as long as 3 months, with some proceeding to become long-term asymptomatic carriers [1]. These transient or chronic carriers can be sources of infection within households [139,140]. In contrast, household members who are well informed of such cases are more likely to acquire knowledge about the disease, its transmission routes, and effective preventive measures. Recent contact with patients with typhoid has also been observed as a potential risk factor, further emphasizing the role of household-level awareness in reducing transmission [48,88]. In many communities, traditional practices of visiting the sick can increase interpersonal contact and inadvertently raise the risk of exposure. Close contacts are often residents of the same area and may share communal water sources, suggesting that transmission could still occur via water contamination (broader environment) rather than direct person-to-person spread. Therefore, health education initiatives should consider addressing the risks associated with visiting infected individuals, alongside broader messaging on water hygiene and disease prevention.

### Hygiene and Sanitation Risk Factors

This study further identified risk factors that underscore the significant impact of hygiene and sanitation on the spread of typhoid fever. Poor handwashing practice is a critical risk factor, given the crucial role hands play in transmitting *S* Typhi through the fecal-oral cycle [26,30,120,123]. While handwashing with soap and clean water effectively removes pathogens [29], inadequacies in technique, such as rinsing without soap [53,122] or neglecting handwashing after defecation [12,97], can increase the risk of bacterial spread [106]. Furthermore, using medicated soaps is an added advantage because it is more effective in eliminating bacteria from hands compared to regular soaps [127].

In addition, the condition of the sewerage system in the house has an important impact on typhoid fever incidence. According to Prasad et al [12], people lacking access to improved sanitation facilities or with damaged improved sewerage systems are particularly vulnerable to infections. In many cases, household toilets are built without professional expertise, often on permeable soil, and in flood-prone areas, increasing the likelihood of leakage and pollution of surface water and crops with human waste [79]. Strengthening the construction and maintenance of sanitary excreta disposal facilities, alongside effective solid waste management, is essential for preventing typhoid fever transmission. Studies have shown that poor sanitation, including improper disposal of solid waste and excreta in residential settings, is directly correlated with higher typhoid prevalence [27,79]. Inadequate waste disposal infrastructure, such as pit latrines, open defecation sites, burst sewer pipes, and the presence of visible feces or urine, has been consistently identified as a significant risk factor [29,53,92,96]. Notably, preventing human excreta from entering the domestic arena has a greater impact on interrupting typhoid transmission than behaviors preventing pathogens in the environment from being ingested by humans (eg, hand washing).

Finally, the discharge of unsterilized water from hospitals and residential areas into rainwater canal systems, often used for irrigating farmlands, contributes to the contamination of vegetables cultivated in these areas [110]. These contaminated crops are frequently consumed without thorough washing, thereby increasing the risk of typhoid fever. Particularly, the risk is pronounced during the rainy season, when heavily polluted irrigation water is more commonly used, and runoff from farms mixed with garbage is more likely to spread into residential zones, further endangering public health.

### Other Risk Factors for Typhoid Fever

Other typhoid risk factors identified in this study include antimicrobial exposure, host-related factors, vaccination status, travel history, and environmental or climate conditions. Among these, antimicrobial use has the greatest impact on *S* Typhi infection. Several studies have shown that prior or recent use of antibiotics, particularly within 4 weeks before disease onset, is associated with an increased risk of typhoid fever, particularly in cases involving multidrug resistant or extensively drug-resistant strains [63,81,101]. Antimicrobial exposure can induce prolonged alterations in gut flora and compromise the barrier against bacterial colonization, thereby reducing the threshold of *S* Typhi required for infection [47]. Studies by Yousafzai et al [119], Srinivasan et al [103], and Kamal et al [81], further highlight antimicrobial resistance as a major contributor to typhoid fever, with certain resistant strains capable of causing epidemics. This resistance is largely caused by the routine presence of *S* Typhi in the human intestine and the indiscriminate use of antibiotics [44,63]. Consequently, drug-resistant *S* Typhi strains, often carrying multiple virulence factors, are becoming increasingly prevalent worldwide. Notably, this study also identified specific risk factors associated with *S* Typhi–resistant strains harboring virulence genes, including those within the H58 lineage, which enhance their ability to infect and interact with host cells [60,62,103,121].

### Host-Related Factors

Host genetic factors influence susceptibility to infectious diseases in humans. This study referenced research by Manal et al [35], which explored the relationship between genetic polymorphisms and typhoid fever risk. Their findings suggested that individuals carrying the 2R3R heterozygote of the intronic variable number tandem repeat in the *IL4* gene may have a genetic predisposition to typhoid fever. However, a study by Dunstan et al [124] reported that a specific haplotype within the tumor necrosis factor gene locus offers protection from typhoid. These associations may be explained by the influence of genetic variation on immune response pathways. Another study [49] found a link between serum anti *H pylori* immunoglobulin G (IgG) levels and an increased risk of typhoid fever. Serum IgG antibodies indicate either prior or active *H pylori* infection, as these antibodies can persist even after infection clearance [49,141,142]. A possible explanation for this association lies in the role of the gastric acid barrier as a crucial defense mechanism against ingested pathogens such as *S* Typhi. *H pylori* infection has been associated with hypochlorhydria, a condition characterized by reduced stomach acid production, which weakens this protective barrier [49,109]. This impairment may facilitate the survival and subsequent colonization of *S* Typhi in the gastrointestinal tract, thereby increasing susceptibility to typhoid fever.

In addition, this review identified a significant association between typhoid fever and the presence of chronic underlying conditions [69]. A plausible explanation is that chronic illnesses can weaken the immune system, impairing the body’s ability to clear *S* Typhi and increasing the risk of persistent or severe infection. For instance, although *S* Typhi is not widely associated with AIDS in developed countries, studies from endemic areas suggest a different pattern. We uncovered a study by Gotuzzo et al [71] that reported an increased risk of typhoid in patients infected with HIV from typhoid-endemic areas. In addition, the study noted that a large proportion of HIV-positive participants were men who have sex with men, raising the possibility that direct fecal-oral transmission may contribute to increased incidence in this subgroup. However, further research is needed to clarify the specific transmission dynamics within this population and to distinguish the role of immunosuppression from that of behavioral factors.

### Travel-Related Risk

Typhoid fever, once prevalent in industrialized countries, is now effectively controlled [91,93]; however, imported infections remain a significant public health concern [39,52,85]. The risk of infection among travelers varies depending on factors such as age, destination, duration, and purpose of travel [99]. Travelers visiting friends or relatives are in a high-risk category for typhoid fever [39,61,73]. As they are much less likely than other travelers to seek pretravel counseling, they may visit more rural, remote areas and engage closely with local people as well as eat high-risk foods and beverages [51]. Children and young adolescents who are visiting friends or relatives are also at high risk of contracting typhoid fever due to a lack of immunity or the possibility of traveling under unhygienic conditions [61,82,99]. We also discovered that traveling to endemic locations increases the risk of contracting typhoid [52,59,86,93]. According to Lin et al [86], more than half of all travelers with typhoid returning to developed countries have visited Asia or Africa, where the disease is widespread. This trend likely reflects increased exposure associated with travel to these endemic areas, particularly during extended stays. In contrast, short-term visitors to endemic areas face a comparatively lower risk of infection [39]. Furthermore, increasing global mobility driven by economic globalization has facilitated the movement of travelers for business, tourism, or labor migration, thereby contributing to disease spread. This growing influx of individuals, often without adequate vaccination or awareness of preventive measures, contributes to the continued transmission and global spread of typhoid fever [82,102].

### Vaccination

Vaccination is essential for the control of typhoid fever in endemic and epidemic settings as well as among travelers moving between nonendemic and endemic areas. The WHO recommends the programmatic use of typhoid fever vaccines in endemic areas [4]. We retrieved 2 studies [75,106] that demonstrated a reduced risk of typhoid fever among individuals who received effective typhoid vaccines and resided in households with improved water. Conversely, poor vaccination coverage, particularly when combined with inadequate WASH infrastructure, can exacerbate typhoid transmission in a given area [63,103]. Moreover, the effectiveness of vaccination may also be compromised by factors, including defective vaccine batches, incorrect immunization procedures, or the interval since vaccination. Evidence from this study suggests that individuals vaccinated more than 3 years prior may face a heightened risk of infection, likely due to waning immunity [94]. This is particularly relevant for polysaccharide vaccines, which have an estimated cumulative efficacy of approximately 55% over 3 years, with the strongest protection occurring within the first 2 years after immunization.

### Environmental, Seasonal, and Climate Factors

Typhoid fever transmission exhibits distinct seasonal patterns influenced by environmental and climatic factors such as temperature, humidity, and precipitation. This study identified diverse peak periods across different endemic regions. For example, Taiwan experiences a surge in cases during the fall (September-November) and winter seasons (December-February) [86], whereas in India, the peak occurs in June in Allahabad [25], and during the monsoon season (July-November) in Ahmedabad [77]. A study by Corner et al [56] discovered that approximately half of the yearly typhoid cases in the Dhaka Metropolitan Area, reaching up to 11 per 100,000 individuals, occurred during summer and fall (July-October). Similarly, Srinivasan et al [103] found a positive correlation between summer temperatures (June-August) and increased typhoid cases. These seasonal fluctuations may stem from a complex interplay of climatic conditions, hygiene practices, and local cultural dynamics [86,143]. In warmer climates or during summer, elevated temperatures enhance the proliferation of *S* Typhi in contaminated foods [103]. Conversely, in colder and more humid conditions, *S* Typhi survive longer in water and soil, thereby increasing the likelihood of environmental transmission [144]. In addition, heavy rainfall during the rainy season can trigger flooding and sewage overflows, leading to contamination of water sources and an increase in typhoid incidences [25,66]. This risk is particularly high in low-lying or flood-prone areas where surface water is commonly used for drinking, cooking, and cleaning [42,56,57]. Settlements in hydrologically vulnerable terrains, such as river floodplains, face particularly heightened risks during these periods due to increased sewage runoff and widespread contamination of water and food supplies [67,78].

### Study Limitations

This study has some limitations. First, publication bias may be present, as studies with significant findings are more likely to be published, potentially underrepresenting negative or null results and skewing risk factor assessments. Second, the lack of formal risk-of-bias assessments may also affect the reliability of findings. In addition, excluding gray literature and non-English studies may have limited the scope, as multilingual and unpublished data could provide further insights. Furthermore, although the review highlights sex-based differences in susceptibility to typhoid fever, the underlying mechanisms remain unclear due to mixed findings, making the proposed explanation, such as occupational exposure in men versus caregiving roles in women, speculative.

### Conclusions

This review combines current knowledge of typhoid fever risk factors and identifies critical areas for targeted intervention. While food and water have been traditionally recognized as the primary transmission pathways for typhoid fever, this review identifies demographic and socioeconomic factors as predominant drivers for transmission. This shift in understanding underscores the need to move beyond conventional mitigation strategies and adopt targeted interventions that address demographic and socioeconomic disparities, which may play a more significant role in typhoid transmission than previously acknowledged. Considering all identified risk factors, mitigation strategies should be prioritized based on regional transmission dynamics and resource availability. In urban Asia, street food regulation should take precedence, while in rural Africa, investment in water infrastructure is equally critical. A combined approach integrating WASH and vaccination programs, particularly in schools and high-risk communities, remains a key priority for long-term disease control in endemic regions. In addition, addressing environmental and climate-related risks, such as flooding and poor waste management, should be incorporated into prevention strategies.

While this review provides a broad synthesis, key knowledge gaps remain. The absence of longitudinal studies limits the understanding of causality and the temporal dynamics of typhoid risk factors. Methodologically, while this review allows a flexible and wide-range synthesis, it lacks the rigor of systematic reviews and meta-analyses, particularly in terms of risk-of-bias assessments and standardized inclusion criteria.

Future studies should focus on longitudinal and case-control methodologies to establish causality in typhoid risk factors, particularly regarding sex, occupation, and disease susceptibility. In addition, structured bias evaluations and quantitative meta-analyses should be incorporated where possible to improve the reliability and comparability of findings. Future research should also investigate the impact of demographic, socioeconomic, and climate variability on typhoid transmission dynamics, given their increasing relevance. Moreover, TCVs are an important tool in reducing *S* Typhi transmission and should be prioritized for introduction in endemic areas to strengthen prevention and control efforts.

By addressing these gaps and priority key areas, future research can strengthen the empirical foundations for typhoid control strategies, enabling policy makers and public health professionals to develop more targeted, evidence-based interventions for effective typhoid fever prevention and control.

## Appendix

Multimedia Appendix 1 PRISMA (Preferred Reporting Items for Systematic Reviews and Meta-Analyses) checklist.

Multimedia Appendix 2 Data extraction entities for the systematic review of typhoid fever risk factors.

Multimedia Appendix 3 Summary of study characteristics and query strings for article search.

## Abbreviations

PRISMA: Preferred Reporting Items for Systematic Reviews and Meta-Analyses
TCV: typhoid conjugate vaccine
WASH: water, sanitation, and hygiene
WHO: World Health Organization
