# Supplementary material for: Risk Factors for Typhoid Fever: Systematic Review
Source: JMIR Public Health Surveill. 2025 Aug 28;11:e67544. doi: 10.2196/67544 (PMC12426575; doi:10.2196/67544)
Supplement: Multimedia Appendix 3 [file publichealth_v11i1e67544_app3.docx]

# **Supplementary information 1**

Supplement to: Risk factors for Typhoid Fever, A desk review

**Table 1. Summary of study characteristics**

| **Characteristics** | **No. of articles (n)** | **Percent (%)** |
| --- | --- | --- |
| **Age groups** |  |  |
| ≤ 15 years | 14 | 13.6 |
| Mixed ages | 83 | 80.6 |
| > 15 years | 6 | 2.8 |
| **Total** | **103** | **100** |
| **Diagnostics** |  |  |
| Culture | 92 | 84.4 |
| Widal and other sero tests | 10 | 9.2 |
| PCR and Sequencing | 7 | 6.4 |
| **Total** | **109** | **100** |
| **Continents** |  |  |
| Africa | 26 | 23.9 |
| Asia | 51 | 46.8 |
| South America | 1 | 0.9 |
| North America | 9 | 8.3 |
| Europe | 11 | 10.1 |
| Australia | 7 | 6.4 |
| mixed continents | 3 | 2.8 |
| undefined | 1 | 0.9 |
| **Total** | **109** | **100** |
| **Typhoid fever cases** |  |  |
| Total count | 253,951 |  |
| Median (IQR) | 110 (51, 283) |  |

**Table 2. Distribution of common typhoid fever risk factors stratified by transmission routes in 108 articles**

| **Transmission route** | **Common factors** | **No. of articles (n)** | **Percent (%)** |
| --- | --- | --- | --- |
| **Waterborne transmission** | |  |  |
|  | Water sources | 10 | 9.2 |
|  | Water supply | 7 | 6.4 |
|  | Contaminated/unsafe water | 10 | 9.2 |
|  | Untreated water | 15 | 13.8 |
|  | Water storage | 3 | 2.8 |
|  |  | **45/109** | **41.3** |
| **Foodborne transmission** |  |  |  |
|  | Street stall/restaurant food | 14 | 12.8 |
|  | Poor food hygiene | 3 | 2.8 |
|  | Food handlers | 8 | 7.3 |
|  | Salads/ other contaminated food | 5 | 4.6 |
|  | Uncooked/raw food | 5 | 4.6 |
|  | Frozen food | 6 | 5.5 |
|  | Milk products | 2 | 1.8 |
|  | Local/traditional food | 2 | 1.8 |
|  | Others | 3 | 2.8 |
|  |  | **48/108** | **44.0** |
| **Socio-economic and housing factors** | |  |  |
|  | Demographic background | 19 | 17.4 |
|  | Socio-economic status | 13 | 11.9 |
|  | Education level | 9 | 8.3 |
|  | Occupational risk | 7 | 6.4 |
|  | Population growth and overcrowding | 6 | 5.5 |
|  | housing system/ condition | 1 | 0.9 |
|  |  | **55/109** | **50.4** |
| **Hygiene and Sanitation factors** | |  |  |
|  | Hygiene and Behavioral factors | 15 | 13.8 |
|  | WASH practices | 3 | 2.8 |
|  | Sanitation and sewage systems | 16 | 14.7 |
|  |  | **34/109** | **31.2** |
| **Others** |  |  |  |
|  | Antimicrobial use | 14 | 12.8 |
|  | Host factors | 6 | 5.5 |
|  | Typhoid carrier/s | 11 | 10.1 |
|  | Vaccination | 6 | 5.5 |
|  | health education | 2 | 1.8 |
|  | Travel-related risk | 19 | 17.4 |
|  | Environmental conditions/factors | 9 | 8.3 |
|  | Climate/ Meteorological factors | 15 | 13.8 |
|  |  | **82/109** | **75.2** |

No. of articles = Number of articles indicating the specific and common factors.

**Query strings for article search**

**Pubmed:** ((risk factors[Title/Abstract]) OR (determinants[Title/Abstract]) OR (predictors[Title/Abstract]) OR (contributors[Title/Abstract]) OR (risk[Title/Abstract]) OR (predisposing factors[Title/Abstract])) AND ((typhoid fever[Title/Abstract]) OR (typhoid[Title/Abstract]) OR (Salmonella typhi[Title/Abstract]) OR (Salmonella typhoid[Title/Abstract]) OR (S. typhi[Title/Abstract]) OR (typhoid disease[Title/Abstract]) OR (typhoidal salmonellosis[Title/Abstract]) OR (typhoidal salmonella[Title/Abstract]))

**Scopus**: TITLE-ABS((risk factors OR determinants OR predictors OR causes OR contributors OR factors OR risk) AND (typhoid fever OR Salmonella typhi OR typhoid OR Salmonella typhoid OR S. typhi OR typhoid disease))

**Semantic scholar**: Risk factors contributors determinants predictors predisposing factor AND typhoid fever typhoid Salmonella typhoid Salmonella typhi S. typhi typhoid disease.

**Google Scholar**: allintitle: Risk factors OR factors OR contributors OR determinants OR predictors OR predisposing risk factors OR risk "typhoid" OR "Salmonella typhi" OR "typhoid fever" OR "S. typhi" "risk factors" -perforation -complication -virulence -severe -non-typhoid
